# Distinguishing Sepsis From Infection by Neutrophil Dysfunction: A Promising Role of CXCR2 Surface Level

**DOI:** 10.3389/fimmu.2020.608696

**Published:** 2020-12-23

**Authors:** Chutima Seree-aphinan, Polathep Vichitkunakorn, Raphatphorn Navakanitworakul, Bodin Khwannimit

**Affiliations:** ^1^ Department of Internal Medicine, Faculty of Medicine, Prince of Songkla University, Songkhla, Thailand; ^2^ Department of Family and Preventive Medicine, Faculty of Medicine, Prince of Songkla University, Songkhla, Thailand; ^3^ Department of Biomedical Sciences, Faculty of Medicine, Prince of Songkla University, Songkhla, Thailand; ^4^ Division of Critical Care Medicine, Department of Internal Medicine, Faculty of Medicine, Prince of Songkla University, Songkhla, Thailand

**Keywords:** apoptosis, CCR2, CD64 index on neutrophils, CXCR2, NEtosis, neutrophils, infection, sepsis

## Abstract

Sepsis is one of the well-established diseases with specific patterns of neutrophil dysfunctions. Previous studies demonstrated sepsis-related neutrophil dysfunctions in comparison with subjects without infection. Since sepsis and infection are recently recognized as distinctive processes, whether these neutrophil dysfunctions are associated with sepsis or infection are not known. Therefore, we longitudinally compared neutrophil functions, widely-cited as exhibiting sepsis-related changes, between patients with septic shock and infection. The surface level of cluster of differentiation 64 (CD64), C-C motif chemokine receptor 2 (CCR2), C-X-C motif chemokine receptor 2 (CXCR2); apoptosis; and NETosis were measured from peripheral blood neutrophils for seven consecutive days using flow cytometry. The between-group comparisons of neutrophil functions were made both on a day-by-day basis and as linear regression between time and measured neutrophil functions (sepsis status included as model predictors). Our study found that, among neutrophil functions studied, only CXCR2 surface level is associated with sepsis. At disease onset, CXCR2 level decrease, with a dose-response relationship with clinical severity. Its level reverts to resemble infected patients by the end of the week. The relationship between CD64 surface level, CCR2 surface level, NETosis, and sepsis are mediated through the effect of infection. Apoptosis activity between these groups are similar, hence, not sepsis-related.

## Introduction

Sepsis is a syndrome diagnosed by the presence of life-threatening organ dysfunctions triggered by infection (1); not all patients with infection develop sepsis. At present, physicians rely on clinical tools [e.g., Sequential Organ Failure Assessment (SOFA) score] to distinguish sepsis from infection, even though signs and symptoms of sepsis overlap considerably with other diseases. As part of an updated sepsis definition (1), it was proposed that dysregulated immunological responses could be the key to distinguish sepsis from an appropriate reaction to infection. Neutrophils could have great potential in this matter since it is considered to have an integral role in sepsis pathobiology; both qualitatively and quantitatively (2). Previous studies consistently identified specific alterations of neutrophil function in sepsis patients (3–5); some of them were linked to poor clinical outcomes (6, 7).

Currently, some knowledge gaps limit a clinical application of these sepsis-related neutrophil dysfunctions. Firstly, most studies demonstrated them in comparison with subjects with good health (8) or pre-existing sterile inflammation (9, 10). In the real clinical setting, however, all sepsis patients are infected. Hence, by using controls without infection, it is impossible to know whether these so-called sepsis-related neutrophil dysfunctions represent a state of infection, infection in conjunction with other inflammatory processes, or sepsis. Secondly, with an update of sepsis definition by the Third International Consensus Definitions for Sepsis and Septic shock (Sepsis-3) (1), neutrophil dysfunctions defined on the background of previous clinical definition may not be relevant to the current practice. Lastly, the paucity of longitudinal studies in this field makes it difficult to generalize the neutrophil dysfunctions demonstrated during one period to another during the clinical course.

Given the knowledge gaps above, our study aims to longitudinally compare neutrophil functions, widely-cited as exhibiting sepsis-related changes, between two groups of patients; clinically defined by Sepsis-3 definition as septic shock or infection. These include the surface level of cluster of differentiation 64 (CD64), C-C motif chemokine receptor 2 (CCR2), C-X-C motif chemokine receptor 2 (CXCR2); apoptosis; and NETosis. The associations between these neutrophil functions and clinical outcomes were also explored.

## Materials and Methods

### Study Design and Participants

This is a longitudinal observational study conducted in a tertiary university teaching hospital in Thailand. Patients who were diagnosed with acute infection and presented within 48 h of their symptom onset were recruited. We used Sepsis-3 definition (1) to classify patients’ sepsis status. Based on these clinical definitions, patients with infection, quick SOFA (qSOFA) score ≥ 2, and evidence of organ dysfunction (SOFA score ≥ 2) are diagnosed as sepsis. Sepsis patients are further categorized as septic shock if, despite adequate fluid resuscitation, they require vasopressor support to maintain mean arterial pressure ≥ 65 mmHg and had serum lactate level > 2 mmol/L. Patients with infection and qSOFA score < 2 are categorized as infection. In our study, only ones who could be classified into either septic shock or infection were included. We did not recruit sepsis patients without septic shock because their clinical signs often overlap with other conditions (e.g., acute heart failure precipitated by infection, acute pulmonary embolism); which potentially lead to misclassification. Patients with less than 18 years of age, prior diagnosis of sepsis within three months before the recruitment, autoimmune diseases, the use of immunosuppressive medications, prolonged steroid usage (> 2 weeks), active malignancy, HIV infection, and pregnancy were excluded. All patients with septic shock were admitted to a medical intensive care unit while patients with infection were admitted to general medical wards. qSOFA score was monitored regularly for patients with infection to ensure their non-sepsis status throughout the study period. All patients received appropriate treatment as outlined in the Surviving Sepsis Campaign Bundles (11). The patients or their legal guardians received verbal informed consent initially followed by written informed consent within 48 h of the study recruitment. Ethical clearance was approved by the Human Research Ethics Committee of the Faculty of Medicine, Prince of Songkla University, Thailand (REC. 61-090-14-1).

### Sample Size Consideration

Our study employed multilevel analyses to explore the longitudinal trends of neutrophil functions and their associations with sepsis; repeated measurements of neutrophil functions were nested within each patient. Hence, group size for multilevel analysis is the number of patients. In medical research, a consensus method of selecting group size for this type of analysis is lacking, even though bigger group size is traditionally believed to give better estimates than the smaller one. Maas et al. (12) conducted a simulation study to evaluate the performance of two-level models, with one explanatory variable in each level, across different group sizes. They found that, compared to the group sizes of 50 and 100, a group size of 30 gives accurate regression coefficients and variance components with negligible bias and only 1.6% higher non-convergence rate. Therefore, we selected a sample size of 30 for our study. During the analysis, we did not encounter any convergence problem.

### Measurement of Neutrophil Functions

For all study participants, two milliliters of fresh whole blood were collected once daily for seven consecutive days (Day 1 to Day 7). Since a clinical course of some septic shock patients may be longer than infected patients, we also collected one additional blood sample at Day 14 in septic shock patients. The surface level of CD64, CCR2, and CXCR2 receptors; apoptosis activity; and NETosis activity were measured from peripheral blood neutrophils using flow cytometry. The degree of CD64, CCR2, and CXCR2 surface level, were quantified by median fluorescent intensity (MFI) of the corresponding molecules in the overall neutrophil population. Additionally, for CD64, CCR2, and CXCR2 receptors, we also quantified the percentages of neutrophils which could be categorized as dysfunctional according to the current literature. Healthy neutrophils have a negligible level of CD64 and CCR2 surface receptors (13, 14) while having abundant CXCR2 surface receptors (15). Compared to normal neutrophils, previous studies have proposed the following neutrophil dysfunctions as sepsis-related: increased phagocytic activity through upregulation of CD64 receptors and transmigration abnormalities due to upregulation of CCR2 receptors or internalization of CXCR2 receptors (3–5). Therefore, gated using unstained neutrophils as negative control, we quantified the percentages of CD64-positive neutrophils, CCR2-positive neutrophils, and CXCR2-negative neutrophils and referred to them as “dysfunctional neutrophils”. Both MFI and the percentages of “dysfunction neutrophils” were used for analyses. Regarding apoptosis and NETosis, the percentages of neutrophils expressing features of apoptosis and NETosis were used to represent apoptosis and NETosis activity.

#### Specimen Collection and Preparation for Flow Cytometry

The blood samples, stored in ethylenediaminetetraacetic acid-coated tubes at room temperature, were processed within 4 h of collection. Neutrophils were isolated by negative selection immunomagnetic cell separation methods using EasySep™ Direct Human Neutrophil Isolation kit (STEMCELL™ Technologies, BC, CA). Fc receptor blockage was carried out as part of the cell separation process. The cell suspensions were separated for two assays, each resuspended in 50 µl of two different pre-mixed antibody cocktails; one cocktail used in the measurement of CD64, CCR2, and CXCR2 surface level and the other used for apoptosis and NETosis studies. Fluorescent-dye conjugated antibodies used in this study are anti-human CD45-Per-CP antibody (BD Pharmingen, NJ, US); anti-human CD16-PE-Cy™7 antibody (BD Pharmingen, NJ, US); anti-human CD64-PE antibody (BD Pharmingen, NJ, US); anti-human CCR2-Alexa Fluor^®^647 antibody (BD Pharmingen, NJ, US); anti-human CXCR2-FITC antibody (BD Pharmingen, NJ, US), Annexin V-FITC antibody (BD Pharmingen, NJ, US); Propidium iodide (PI) (BD Pharmingen, NJ, US); and anti-human myeloperoxidase (MPO)-PE antibody (Bio-Rad Laboratories, CA, US). The samples were incubated for 20 min in the dark at room temperature before fixing with 1% paraformaldehyde solution for 4 min. The cells were resuspended in Dulbecco’s phosphate-buffered saline and kept in the dark, waiting for data acquisition within 4 h of fixation. The purity of the neutrophil isolates was evaluated in all experimental specimens by CD45/Side scatter gating. All experiments conducted in this study achieved more than 95% of neutrophil purity.

#### Flow Cytometry Analysis

Data acquisition was performed with the Amnis^®^ ImageStream^®^X Mk II Imaging Flow Cytometer (Luminex Corporation, TX, US). The fluidics was set at low flow with high sensitivity and 40X magnification objective. In addition, 488- and 642-nm lasers with an output power of 100 and 150 mW were used. We followed Maecker et al. (16) recommendations for the flow cytometry control set-up. We used background fluorescent intensities from unstained cells as negative control. Neutrophils from two septic shock patients were used for positive control experiments. The optimal antibody concentration which gave positivity while minimizing image oversaturation was titrated. The positivity was confirmed both by comparing the fluorescent intensity with unstained samples and the visualization of the surface staining on fluorescent cell images, which were simultaneously collected by the Imaging Flow Cytometer. For positive control of apoptosis, neutrophils were cultured in Roswell Park Memorial Institute 1640 Medium (ThermoFisher Scientific, MA, US), supplemented with 10% heat-inactivated fetal bovine serum (ThermoFisher Scientific, MA, US) and 1× Gibco^®^ Antibiotic-Antimycotic (ThermoFisher Scientific, MA, US) at 37°C, CO2 5%, for 48 h before analysis to allow time for most of the cells to undergo apoptosis. For positive control of NETosis, we used 1× concentration of eBioscience™ Cell Stimulation Cocktail (ThermoFisher Scientific, MA, US), a mixture of phorbol 12-myristate 13-acetate (PMA) and ionomycin, to stimulate NETosis formation. Both PMA and ionomycin are well-known NETosis inducers (17).

**Figure 1:**
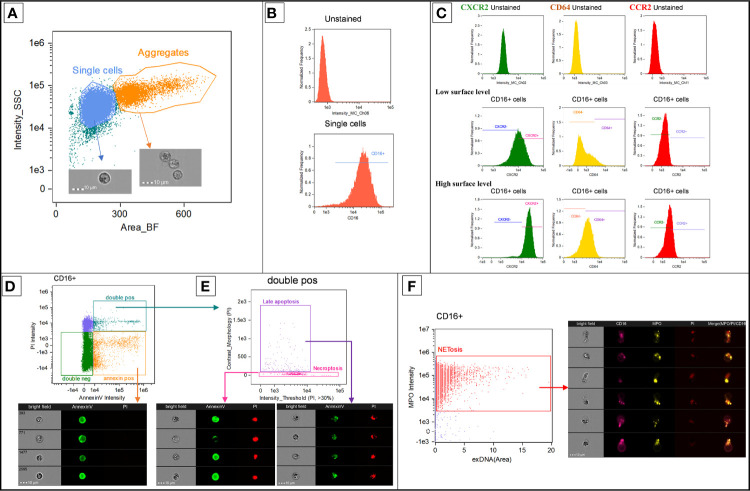
Representative diagrams showing the flow cytometry gating strategy. This analysis was performed on IDEAS^®^ software version 6.2. Propriety software functions are italicized. Prior to analysis, the collected events underwent camera focusing quality assessment using *Gradient RMS* and, for the assay which measured CD64, CCR2, and CXCR2 surface level, dead cells elimination. The selected events were firstly gated on brightfield *Area* and side scatter intensity to evaluate their size and granularity, respectively **(A)**. Single cells were separated from cell aggregates by brightfield *Area* (labeled as Area_BF) and preliminarily identified as neutrophils based on their granularity. CD16 positivity was used to confirm the gated cells as neutrophils **(B)**. The MFI of CD64, CCR2, and CXCR2 surface receptors was measured from neutrophils; which were also gated as positive or negative population using unstained samples as negative control **(C)**. Using Annexin-V and PI intensity, CD16-positive cells were distinguished into double-negative (healthy) cells, Annexin V-positive/PI-negative (early apoptotic) cells and double-positive (late apoptotic and necroptotic) cells **(D)**. *Intensity threshold* and *Contrast morphology* for the PI-channel were employed to discriminate between late apoptotic and necroptotic cells **(E)**. A co-staining of MPO and cell-appendant extracellular DNA was used to identify neutrophils undergoing NETosis **(F)**. The area of extracellular DNA, labeled as exDNA (Area), was established by subtracting BF mask from PI-channel mask; since BF and PI-channel masks delineate cell boundaries and DNA extent, respectively. CCR2, C-C chemokine receptor 2; CD, cluster of differentiation; CXCR2, C-X-C chemokine receptor 2; DNA, Deoxyribonucleic acid; MFI, Median fluorescent intensity; MPO, myeloperoxidase; PI, propidium iodide; SSC, side scatter.

Image Data Exploration and Analysis Software (IDEAS^®^) version 6.2 was used for flow cytometry analysis. Propriety software functions are italicized. Spectral overlap compensation was performed by using single stained controls to create a compensation matrix. Prior to analysis, the collected events underwent camera focusing quality assessment using the software’s function called *Gradient RMS* and included only events with good camera focus. For the assay which measured CD64, CCR2, and CXCR2 surface level, dead cell (i.e., PI-positive events) elimination was also performed. The selected events were firstly gated on brightfield (BF) *Area* and side scatter intensity to evaluate their size and granularity, respectively (**Figure 1A**). Single cells were separated from cell aggregates by objects’ size quantified by the software’s image-based analytic function called *Area*. This function measures the size of the object using image pixels. By applying this function to the BF images, this value represents the cross-sectional area of the object images (i.e., the size of the cell). Utility-wise, it is equivalent to forward scatter geometry in non-imaging flow cytometers. Among single cells, neutrophils were preliminarily identified based on their granularity. Subsequently, CD16 positivity was used to confirm the gated cells as neutrophils (**Figure 1B**); every experimental specimen yielded at least 10,000 neutrophils for further analyses. On CD16-positive neutrophils, we measured the MFI of CD64, CCR2, and CXCR2 receptors, as well as the percentages of CD64-positive cells, CCR2-positive cells, and CXCR2-negative cells (**Figure 1C**). Regarding apoptosis cell count (**Figures 1D, E**), we used a method described by Pietkiewicz et al. (18), which combines an image-based flow cytometry analysis and classical Annexin V/PI staining for apoptosis detection. The method distinguishes cell population into double-negative (healthy) cells, Annexin V-positive/PI-negative (early apoptotic) cells, and double-positive (late apoptotic and necroptotic) cells. Among the double-positive population, the software’s image-based analytic functions, namely *Intensity threshold* and *Contrast morphology* for the PI-channel, were employed to discriminate between late apoptotic and necroptotic cells. We collectively counted early and late apoptosis as apoptosis events. NETosis was identified by the presence of cell-appendant neutrophil extracellular traps components, including MPO and extracellular DNA. Extracellular DNA was detected by using the software masking features as previously described (19). These masking features identify cells with DNA contents located extracellularly and help exclude the cells stained by PI intracellularly (late apoptotic and necroptotic cells). In brief, a cell-impermeant DNA dye, PI in our experiment, was used to stain extracellular DNA. The software creates masks based on the fluorescent images of the cells stained by PI. The area of extracellular DNA was established by subtracting BF mask from PI-channel mask; since BF and PI-channel masks delineate cell boundaries and DNA extent, respectively. Neutrophils with high extracellular DNA area and MPO positivity were counted as NETosis events (**Figure 1F**).

### Data Collection

The following baseline clinical data were collected: age, gender, comorbidities, source of infection, presence or absence of bacteremia, initial absolute neutrophil count, Acute Physiology and Chronic Health Evaluation (APACHE) II score on admission, and patient’s status at Days 7, 14, and 28 (i.e., recovered, critically-ill, deceased). Patients were considered critically-ill if they still needed organ support (e.g., mechanical ventilation, vasopressors). Survivors, who did not fall into the critically-ill definition, were classified as recovered. Daily SOFA scores were collected as means to quantify clinical severity in septic shock patients.

### Statistical Analysis

Data analysis was performed using STATA 16.1 (StataCorp LLC, TX, US). The graphical illustrations were created with the R program’s data visualization package (ggplot2). The baseline characteristics of patients between septic shock and infection groups were compared with Pearson’s chi-squared or Fisher’s exact tests as appropriate. The following measurements of neutrophil functions were used in the analyses: MFI of CD64, CCR2, and CXCR2 surface receptors; the percentages of “dysfunction neutrophils” (i.e., CD64-positive neutrophils, CCR2-positive neutrophils, and CXCR2-negative neutrophils); the percentages of apoptosis; and the percentages of NETosis. For measurements of CD64, CCR2, and CXCR2 surface receptors, pairwise correlation analyses were performed to determine the degree of correlations between MFI and the percentages of “dysfunction neutrophils”. The analyses of associations between each neutrophil function and sepsis status were divided into two parts as described below with p < 0.05 defined statistical significance of all tests.

#### Longitudinal Changes of Neutrophil Functions and Their Associations With Sepsis Status

The comparison of neutrophil functions between septic shock and infected patients was made both on a day-by-day basis and as linear regression between time (i.e., days after admission) and measured neutrophil functions. The measurements of neutrophil functions on specific days were compared with t-tests and Wilcoxon Rank Sum tests for normally and non-normally distributed variables, respectively. Additionally, the measurements of neutrophil functions on Day 7 and Day 14 in the septic shock group were compared using paired t-tests or Wilcoxon matched-pair signed-rank tests as appropriate.

Multilevel regression analyses were performed to examine associations between the longitudinal changes of each neutrophil function and sepsis status while simultaneously explore the degree of interindividual variation in the response patterns. We used two-level mixed linear models with repeated measurements of neutrophil functions (level-1 variable) nested within each patient (level-2 variable). Fixed portion of the models determined the relationship between time (i.e., days after admission) and the measurements of each neutrophil functions. Random portion of the model, also calculated as intraclass correlation coefficients conditioned on a level-2 group effect, represented the degree of interindividual variation of the response patterns. Associations between sepsis status and the longitudinal changes of each neutrophil function were assessed by adding patient’s sepsis status (i.e., septic shock, infection) and its interaction with time as model predictors. This model structure aimed to estimate the effect of patient’s sepsis status in predicting the changes of neutrophil functions after adjusting for effects of time and interindividual variation. We hypothesized that both infection and septic shock may exert effects on the neutrophil functions, neither one of them should be the base level of the other during regression analyses. Hence, we included both infection status and septic shock status into the model so that their regression coefficients can be compared. The models were executed on the restricted maximum likelihood and the Kenward-Roger degrees of freedom methods, and unstructured covariance between the model’s intercept and slope was allowed.

#### Associations Between Neutrophil Functions and Clinical Outcomes

The clinical outcomes of interest composed of Day 14 clinical status (i.e., recovered, critically-ill, deceased) and, in the subgroup of septic shock patients, SOFA score. Depending on data distribution, either one-way analysis of variance (ANOVA) tests or Kruskal Wallis tests with Dunn’s test of multiple comparison was employed to compare the measurements of each neutrophil function on specific days among patients stratified by Day 14 clinical status. Moreover, in septic shock patients, we used the multilevel analysis, structured as described above, to estimate the associations between the measurements of each neutrophil function and SOFA score.

## Results

### Patient Characteristics

We included 19 septic shock patients and 11 infected patients into the study (**Table 1**). Overall, our study population was elderly patients with many comorbidities. The study participants with septic shock and infection were similar in age, gender, and comorbidities. The difference between them was their clinical status; septic shock patients had a more severe clinical picture as demonstrated by APACHE II score and the percentages of patients who were critically-ill or deceased at Days 7, 14, and 28. Six patients were lost to follow-up toward the end of the specimen collection period resulting in 14 missing observations (6.5% of the expected number of observations). The number of longitudinal observations per patient ranged from 3 to 8, with a median of 7.

**Table 1:** Characteristics of study participants^†^ (n = 30).

Characteristics	Septic shock (n = 19)	Infection(n = 11)	p-value
Age (median, 95%CI)	74 (70–83)	78 (70–81)	0.93^1^
Female (%)	42	45	0.58^1^
Co-morbidities (%)			
Cardiovascular disease	47	45	0.92^2^
Diabetes mellitus	42	27	0.34^1^
Neurological disease	32	45	0.35^1^
Respiratory disease	26	18	0.49^1^
Liver disease	21	0	0.14^1^
Renal disease	16	27	0.38^1^
Hematologic disease	11	9	0.70^1^
Source of infection (%)			0.87^1^
Respiratory tract	32	36	
Gastrointestinal tract	26	36	
Urinary tract	21	18	
Others	21	9	
Bacteremia (%)	53	18	0.07^1^
Initial absolute neutrophil count [×10^9^/L] (median, 95%CI)	15,020(12,994–21,071)	11,011(10,380–18,403)	0.29^3^
APACHE II score (mean, 95%CI)	29 (25–32)	13 (9–17)	<0.001*^4^
Patient’s status 7 days after recruitment (%)			0.004*^1^
Recovered	42	100	
Critically ill	37	0	
Deceased	21	0	
Patient’s status 14 days after recruitment (%)			0.008*^1^
Recovered	32	91	
Critically ill	47	9	
Deceased	21	0	
Patient’s status 28 days after recruitment (%)			0.008*^1^
Recovered	42	100	
Critically ill	32	0	
Deceased	26	0	

^†^All study participants were diagnosed with acute infection and presented within 48 h of their symptom onset.

*p value < 0.05.

^1^Fisher’s exact test ^2^Pearson’s chi-squared test ^3^Wilcoxon Rank Sum test ^4^t-test.

APACHE II, Acute Physiology and Chronic Health Evaluation II; CI, confidence interval.

### Comparison of Neutrophil Functions Between Septic Shock and Infected Patients

**Figure 2:**
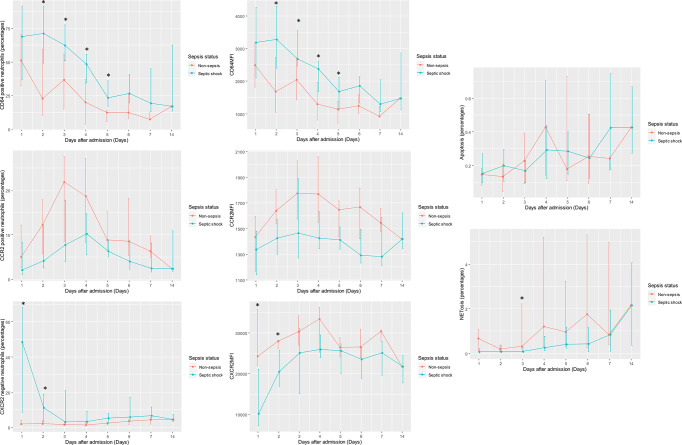
Daily measurements of CD64, CCR2, and CXCR2 surface level; apoptosis; and NETosis stratified by sepsis status (septic shock or infection). MFI of CD64, CCR2, and CXCR2 surface receptors; the percentages of “dysfunction neutrophils” (i.e., CD64-positive neutrophils, CCR2-positive neutrophils, and CXCR2-negative neutrophils); the percentages of apoptosis; and the percentages of NETosis were illustrated in line graphs with error bars grouped by sepsis status. The lines connected median value of the measurements on the specific days. Error bars delineated interquartile ranges. Depending on data distribution, either t-test or Wilcoxon Rank Sum test was employed to compare the measurements of each neutrophil function on specific days between groups. *p-value from t-tests or Wilcoxon Rank Sum tests < 0.05. CCR2, C-C chemokine receptor 2; CD64, cluster of differentiation 64; CXCR2, C-X-C chemokine receptor 2; MFI, median fluorescent intensity.

Daily measurements of neutrophil functions stratified by sepsis status were shown in **Tables 2** and **3**. They were also illustrated in graphics (**Figure 2**). Regarding CD64, CCR2, and CXCR2 surface levels, pairwise correlation analyses demonstrated strong correlations between MFI and the percentages of dysfunctional neutrophils, defined by the corresponding molecules (correlation coefficients > 0.7). CD64 and CCR2 MFI positively correlated with the percentages of CD64-positive cells and CCR2-positive cells with correlation coefficients of 0.95 and 0.94, respectively. For CXCR2 receptors, there was a negative correlation between the percentages of CXCR2-negative neutrophils and CXCR2MFI with a correlation coefficient of −0.77.

**Table 2:** The daily percentages of CD64-positive neutrophils, CCR2-positive neutrophils, CXCR2-negative neutrophils, apoptosis, and NETosis stratified by sepsis status^†^ (n = 30: septic shock = 19, infection = 11).

Day	%, Median (95%CI)									
	CD64-positive neutrophils		CCR2-positive neutrophils		CXCR2-negative neutrophils		Apoptosis		NETosis	
	Septic shock	Infection	Septic shock	Infection	Septic shock	Infection	Septic shock	Infection	Septic shock	Infection
1	68.90 (37.91–91.32)	51.10 (26.03–78.33)	2.08 (1.02–7.67)	5.00 (2.00–12.90)	48.60*^2^ (8.88–67.53)	2.05 (0.87–6.10)	0.15 (0.10–0.27)	0.15 (0.06–0.20)	0.07 (0.01–0.21)	0.67 (0.06–1.41)
2	71.20*^1^ (50.10–90.96)	22.70 (7.58–60.26)	4.13 (2.49–11.73)	12.20 (3.89–20.71)	11.10*^2^ (3.45–17.75)	2.14 (1.18–5.19)	0.20 (0.11–0.28)	0.13 (0.03–0.23)	0.09 (0.03–0.34)	0.19 (0.03–1.92)
3	62.25*^1^ (48.57–79.17)	36.70 (10.93–63.65)	7.67 (3.29–25.19)	21.80 (7.97–29.73)	3.22 (1.59–28.80)	1.69 (0.88–3.33)	0.17 (0.09–0.25)	0.23 (0.08–0.48)	0.09 (0.02–0.30)	0.32*^2^ (0.14–5.47)
4	48.50*^1^ (34.50–56.19)	19.90 (3.27–40.09)	10.20 (5.24–15.97)	18.70 (2.93–30.11)	3.35 (2.13–9.81)	1.52 (0.79–3.30)	0.29 (0.12–0.49)	0.43 (0.04–0.78)	0.25 (0.09–1.00)	1.20 (0.16–9.71)
5	23.25*^2^ (12.02–39.93)	12.50 (3.91–14.86)	6.28 (4.68–9.82)	8.85 (5.09–16.89)	5.37 (2.25–11.82)	2.39 (1.16–4.25)	0.29 (0.11–0.50)	0.18 (0.09–0.91)	0.41 (0.16–1.36)	0.95 (0.16–5.58)
6	26.50 (17.07–41.28)	12.35 (3.70–33.54)	4.00 (1.73–11.93)	8.54 (3.98–20.97)	5.91 (3.49–25.27)	3.54 (1.20–6.17)	0.24 (0.10–0.57)	0.25 (0.07–0.76)	0.42 (0.05–1.42)	1.76 (0.10–6.27)
7	19.30 (7.62–61.10)	7.44 (3.65–19.43)	2.45 (1.14–10.43)	6.32 (2.27–22.68)	6.69 (3.02–12.36)	4.32 (0.99–8.04)	0.43 (0.15–1.38)	0.24 (0.12–0.27)	0.84 (0.06–2.84)	0.84 (0.03–8.66)
14	17.00 (8.96–86.80)	N/A	2.38 (1.52–17.53)	N/A	4.46 (1.91–14.29)	N/A	0.43 (0.17–0.88)	N/A	2.16*^3^ (0.08–4.67)	N/A

^†^Neutrophil functions, including the percentages of “dysfunction neutrophils” (i.e., CD64-positive neutrophils, CCR2-positive neutrophils, and CXCR2-negative neutrophils); the percentages of apoptosis; and the percentages of NETosis, were measured from peripheral blood neutrophils daily for seven consecutive days using flow cytometry. One additional blood sample at Day 14 was collected in septic shock patients to cover their more prolonged clinical courses. T-tests or Wilcoxon Rank Sum tests were applied to compare the measurement of neutrophil functions between septic shock and infection groups, as appropriate. In septic shock groups, Wilcoxon matched-pairs signed-rank tests were used to compare the measurement of neutrophil functions between Day 7 and Day 14.

*1 p-value < 0.05 from t tests, compared with infection group.

*2 p-value < 0.05 from Wilcoxon Rank Sum tests, compared with infection group.

*3 p-value < 0.05 from Wilcoxon matched-pairs signed-rank tests, compared with Day 7 in the septic shock group.

CCR2, C-C chemokine receptor 2; CD64, cluster of differentiation 64; CI, confidence interval; CXCR2, C-X-C chemokine receptor 2; N/A, not applicable.

**Table 3:** MFI of CD64, CCR2, and CXCR2 surface receptors stratified by sepsis status^†^ (n = 30: septic shock = 19, infection = 11).

Day	MFI, Median (95%CI)					
	CD64		CCR2		CXCR2	
	Septic shock	Infection	Septic shock	Infection	Septic shock	Infection
1	3193.94 (2,145.81, 4,242.25)	2492.23 (1,597.89, 3,313.78)	1,337.01 (1,151.99, 1,450.36)	1,433.08 (1,142.61, 1,697.64)	10,196.71*^1^ (7,445.314, 2,0940.83)	24,298.33 (20,439.08, 39,750.90)
2	3279.44*^1^ (2,435.43, 4,260.54)	1679.51 (618.47, 2,781.14)	1,425.21 (1,302.48, 1,558.88)	1,638.60 (1,483.28, 1,905.19)	2,0549.02*^1^ (17,144.55, 25,486.94)	28,013.80 (24,471.31, 28,631.53)
3	2683.48*^1^ (2,382.53, 3,638.18)	2049.4 (1,103.699, 3,115.549)	1,465.15 (1,237.06, 2,031.36)	1,774.42 (1,545.59, 2,052.88)	25,155.37 (13,073.41, 31,818.24)	3,0382.27 (26,048.04, 35,935.84)
4	2381.37*^2^ (1,657.00, 2,621.17)	1298.55 (529.29, 2,042.85)	1424.52 (1,314.32, 1,686.46)	1,768.58 (1,417.53, 2,085.80)	25,959.55 (23,896.68, 29,663.46)	3,3488.80 (24,209.54, 37,841.40)
5	1677.98*^2^ (1,045.90, 2,255.14)	1139.14 (498.70, 1,417.34)	1,411.16 (1,297.23, 1,564.13)	1,645.81 (1,368.05, 1,744.92)	25,675.21 (18,383.27, 28,491.75)	26,454.04 (22,687.25, 29,959.20)
6	1862.86 (1,316.16, 2,202.05)	1242.69 (801.35, 1,833.44)	1,292.21 (1,170.29, 1,591.30)	1,667.09 (1,374.63, 1,854.49)	23,544.83 (17,297.05, 27,167.73)	26,602.44 (22,541.86, 32,232.45)
7	1297.45 (953.56, 2,778.40)	925.66 (186.11, 1,461.47)	1,282.14 (1,070.26, 1,694.45)	1,546.44 (1,360.41,1971.56)	25,100.66 (17,539.61, 29,322.77)	30,463.56 (17,820.35, 31,059.70)
14	1478.60 (635.125, 4,203.85)	N/A	1,420.93 (1,239.58, 1,932.46)	N/A	21,764.50 (17,396.15, 25,409.94)	N/A

^†^Neutrophil functions, including MFI of CD64, CCR2, and CXCR2 surface receptors, were measured from peripheral blood neutrophils daily for seven consecutive days using flow cytometry. One additional blood sample at Day 14 was collected in septic shock patients to cover their more prolonged clinical courses. T-tests or Wilcoxon Rank Sum tests were applied to compare the measurement of neutrophil functions between septic shock and infection groups, as appropriate. In septic shock groups, paired t-tests or Wilcoxon matched-pairs signed-rank tests were used to compare the measurement of neutrophil functions between Day 7 and Day 14.

*1 p-value < 0.05 from t-tests, compared with infection group.

*2 p-value < 0.05 from Wilcoxon Rank Sum tests, compared with infection group.

CCR2, C-C chemokine receptor 2; CD64, cluster of differentiation 64; CI, confidence interval; CXCR2, C-X-C chemokine receptor 2; MFI, median fluorescent intensity; N/A, not applicable.

Averaged among patients with the same clinical diagnosis, there was a statistically significant difference of CD64 and CXCR2 surface levels as well as NETosis activity between septic shock and infection groups, although the differences could only be demonstrated in some specific days during the clinical course. Neutrophils from septic shock patients had a higher CD64MFI as well as the higher percentages of CD64-positive neutrophils during Day 2 to Day 5. They also showed a lower CXCR2MFI and the higher percentages of CXCR2-negative neutrophils on Day 1 and Day 2. On the contrary, NETosis activity in infected patients was greater than that of septic shock patients on Day 3. In septic shock patients, however, there was a significant increase in NETosis activity from Day 7 to Day 14 in contrast to other neutrophil functions in which the changes in their levels between Day 7 and Day 14 were non-significant. Interestingly, the percentages of NETosis on Day 14 in septic shock patients and Day 3 in infected patients were in a similar range, no statistically significant difference between them can be demonstrated using Wilcoxon Rank Sum tests. Also, no statistically significant difference in CCR2MFI, a percentage of CCR2-positive neutrophils, and apoptosis activity, between septic shock and infection groups was found.

The multilevel regression analyses assessing longitudinal changes of neutrophil functions and their associations with sepsis status were demonstrated in **Table 4**. For CD64, CCR2, and CXCR2 surface levels, since the percentages of dysfunctional neutrophils were highly correlated with MFI and their unit of measurement synchronizes with apoptosis and NETosis, we used the percentages of dysfunctional neutrophils for the regression analysis; so that the coefficients and variances derived from the model of each neutrophil function are comparable. All neutrophil functions studied, except for CCR2 surface level, changed dynamically over time (p-value from Wald’s tests for the model coefficient of time < 0.05). The percentages of CD64-positive neutrophils and CXCR2-negative neutrophils declined over time as opposed to apoptosis and NETosis, which increased over time. The percentages of CCR2-positive neutrophils, however, fluctuated throughout the study period. Some degree of interindividual variation of the magnitude of change existed for all neutrophil functions examined as demonstrated by the model variances and level-2 conditional ICCs. The degree of interindividual variation was largest for CCR2 surface level and smallest for apoptosis activity. Adjusting for the effect of time, septic shock status could predict the longitudinal trends of CD64, CCR2, and CXCR2 surface levels. Infection status could also predict CD64 and CCR2 surface levels over time; albeit lack of ability to predict that of CXCR2 receptors. In contrast, our analyses suggested that the changes in apoptosis and NETosis activity during the study period could only be predicted by time. Furthermore, for the longitudinal trends of CXCR2 surface level, there was a significant interaction between time and sepsis status in which an effect of sepsis status diminishes as time passes. In the regression models involving other neutrophil functions, no statistically significant interaction between time and sepsis status was demonstrated.

**Table 4:** Multilevel mixed model analysis examining the longitudinal correlations between the percentages of CD64-positive neutrophils, CCR2-positive neutrophils, CXCR2-negative neutrophils, apoptotic, and NETosis and sepsis status† (n = 30).

Model parameters		Estimate (95%CI)				
		CD64-positive neutrophils	CCR2-positive neutrophils	CXCR2-negative neutrophils	Apoptosis	NETosis
Fixed effects	Coefficient: Time (*β*_1_)	−5.24* (−7.65, −2.83)	−0.62 (−1.91, 0.67)	−3.18* (−4.87, −1.50)	0.09* (0.02, 0.16)	0.28* (0.09, 0.47)
	Coefficient: Infection (*β*_2_)	51.59* (31.70, 71.48)	15.82* (4.15, 27.49)	0.82 (−14.44, 16.09)	0.22 (−0.13, 0.57)	1.40 (−0.13, 2.94)
	Coefficient: Sepsis shock (*β*_3_)	71.20* (56.11−86.28)	12.99* (4.14, 21.83)	32.29* (20.76, 43.82)	0.11 (−0.15, 0.37)	−0.24 (−1.39, 0.92)
	Interaction term:Time x Sepsis status (*β*_4_)	1.03 (−2.96,5.01)	−0.14 (−2.30, 2.02)	−4.05* (−6.90, −1.20)	0.06 (−0.06,0.17)	−0.06 (−0.38,0.25)
Random effects	$\sigma_{\mu_{0}}^{2}$	848.59 (448.91, 1604.13)	305.02 (164.16, 566.75)	466.97 (231.45, 942.15)	0.02 (0.001,0.53)	3.82 (1.64, 8.89)
	$\sigma_{\mu_{1}}^{2}$	16.18 (7.09, 36.93)	5.08 (2.02, 12.77)	6.26 (1.67, 23.50)	0.01 (0.002, 0.02)	0.04 (0.01, 0.28)
	*σ*_01_	−88.98 (−165.51, −12.46)	−37.14 (−64.86, −9.43)	−49.60 (−98.92, −0.28)	−0.01 (−0.04, 0.01)	0.11 (−0.28, 0.50)
	$\sigma_{e}^{2}$	253.82 (201.50, 319.74)	70.93 (55.71, 90.30)	196.27 (155.57, 247.62)	0.41 (0.34, 0.51)	3.16 (2.51, 3.98)
	Level-2 ICC	0.77 (0.63, 0.87)	0.81 (0.68, 0.90)	0.70 (0.53, 0.84)	0.05 (0.002,0.56)	0.55 (0.33, 0.75)

^†^ Regression equation: PMN_ij_ = β_0_ + β_1_(Time_ij_) + β_2_(Infection_j_) + β_3_(Septic shock_j_) + β_4_(Time_ij_ × Sepsis_j_) + µ_0j_ + µ_1j_(Time_ij_) + e_ij_;

i, measurement occasions; j, individual patients; PMN_ij_, repeated measurements of the neutrophil functions; Time, days after admission (days); Sepsis, patient’s sepsis status (infection or septic shock); β_0_, intercept; β_1_, coefficient of time; β_2_, coefficient of infection (0 = No, 1 = Yes); β_3_, coefficient of septic shock (0 = No, 1 = Yes); β_3_, coefficient of an interaction term between Time and Sepsis; µ_0j_, patient-specific random effects of intercept; µ_1j_, patient-specific random effects of slope for time; e_ij_, occasion-specific time-varying residuals;$\sigma_{\mu_{0}}^{2}$, between-patient intercept variance;$\sigma_{\mu_{1}}^{2}$, between-patient time slope variance; σ_01_, between-patient intercept-slope covariance; $\sigma_{e}^{2}$, within-patient variance.

* p-value < 0.05.

CCR2, C-C chemokine receptor 2; CD64, cluster of differentiation 64; CI, Confidence interval; CXCR2, C-X-C chemokine receptor 2; Level-2 ICC, intraclass correlation coefficient conditioned on the effect of interindividual variation.

### Associations Between Neutrophil Functions and Clinical Outcomes

**Figure 3:**
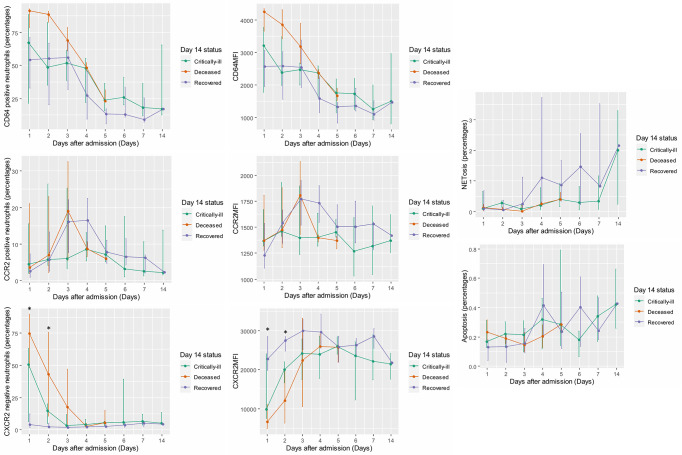
Daily measurements of CD64, CCR2, and CXCR2 surface level; apoptosis; and NETosis stratified by Day 14 clinical status (Recovered, Critically-ill, or Deceased). MFI of CD64, CCR2, and CXCR2 surface receptors; the percentages of “dysfunction neutrophils” (i.e., CD64-positive neutrophils, CCR2-positive neutrophils, and CXCR2-negative neutrophils); the percentages of apoptosis; and the percentages of NETosis were illustrated in line graphs with error bars grouped by Day 14 clinical status. The lines connected median value of the measurements on the specific days. Error bars delineated interquartile ranges. Depending on data distribution, either one-way ANOVA test or Kruskal Wallis test with Dunn’s test of multiple comparison was employed to compare the measurements of each neutrophil function on specific days between groups. *p-value from ANOVA or Kruskal Wallis tests < 0.05. ANOVA, analysis of variance; CCR2, C-C chemokine receptor 2; CD64, cluster of differentiation 64; CXCR2, C-X-C chemokine receptor 2, MFI, median fluorescent intensity.

Daily measurements of neutrophil functions stratified by Day 14 clinical status were illustrated in **Figure 3**. The distinction between patients with varying clinical status can be best appreciated in the longitudinal trends of CXCR2MFI and the percentages of CXCR-negative neutrophils. The statistically significant difference in CXCR2MFI and the percentages of CXCR2-negative neutrophils among three groups (recovered, critically-ill, deceased) can be demonstrated on Day 1 and Day 2. During the first two days of admission, CXCR2MFI were lowest and the CXCR2-negative neutrophils were highest in patients who died within 14 days after admission. Also, the multilevel regression analysis assessing associations between the measurements of neutrophil functions and SOFA score found that, after adjusting for the effect of time, the percentages of CXCR2-negative neutrophils could predict SOFA score in patients with septic shock (p = 0.001). CD64MFI and the percentages of CD64-positive neutrophils seemed to be highest in deceased patients as opposed to NETosis activity which was highest in patients who recovered. Unfortunately, these trends of CD64 surface level and NETosis activity could not be proved statistically significant; either by between-groups comparisons or multilevel regression analysis assessing its association with SOFA score. The longitudinal trends of CCR2 surface level and apoptosis among patients with different Day 14 clinical status were similar.

## Discussion

Numerous physiological and pathological stimuli could trigger changes in neutrophil functions. Various neutrophil phenotypes have been discovered, not only in healthy but also diseased states (20); some with potential for clinical applications (21, 22). Sepsis is one of the well-established diseases resulting in specific patterns of neutrophil dysfunctions; increased CD64 and CCR2 surface levels; decreased CXCR2 surface level; apoptosis delay; and increased NETosis are widely-cited sepsis-related neutrophil dysfunctions. However, the pattern that distinguishes sepsis from infection has not been mentioned; this is partly because sepsis and infection were first recognized and emphasized as distinctive processes in the newest update of sepsis definition (Sepsis-3). Therefore, literature, published before the release of this definition, has been employing non-uniform control settings to establish neutrophil dysfunctions as sepsis-related; none of them used infection as control. Therefore, previously-identified sepsis-related neutrophil dysfunctions are potentially a product of a combined effect between sepsis and infection rather than sepsis itself. However, we hypothesize that, on a background of infection, some of them might be genuinely sepsis-related, if we re-evaluate them in the desirable control setting.

In this study, we compared the neutrophil functions, with reported sepsis-related changes, between patients with septic shock and patients with infection who did not develop sepsis. Since sepsis is a time-dynamic disease, we observed these neutrophil functions longitudinally during the early phase of sepsis. We found that, during this period, each neutrophil function follows differing courses of changes and has a unique relationship with sepsis. Of all neutrophil functions examined by our study, we can only affirm a reduction of CXCR2 surface level as sepsis-related. CXCR2 receptors play a significant role in the normal regulation of neutrophil recruitment (15). Previous experimental studies have demonstrated reduced CXCR2 surface level due to CXCR2 receptor internalization induced by circulating chemokines (15, 23). The lack of CXCR2 surface receptors was also linked to severe neutrophil hyperplasia in the bone marrow and neutrophilia in sepsis mice model (24). In addition, our study found that a reduction of CXCR2 surface level is related to sepsis, even in the presence of infection. Compared to patients with infection, CXCR2 surface level decreases at the onset of the disease in septic shock patients. As shown by the regression models, septic shock status predicts CXCR2 surface level over time while infection status does not. As patients progress into the less dynamic phase of the disease, CXCR2 surface level increases so that, by the end of the week, the difference of CXCR2 surface level between sepsis and infected patients disappears. Additionally, during early sepsis, patients with more severe disease have a significantly lower CXCR2 surface level than those with milder disease. In fact, in infected patients, CXCR2 surface level is persistently high, closely resembled a response pattern of healthy volunteers (8). These findings suggest that a reduction of CXCR2 surface level occurs synchronously with the peak of disease activity; shows the ability to revert to normal state; and has a dose-response relationship with clinical outcomes. Based on our observation, abnormal neutrophil migration due to decreased CXCR2 surface receptors should be considered the immunopathological feature, which could act as a marker for distinguishing sepsis from infected patients at the onset of the disease. As a consequence of decreased CXCR2 surface level, appropriate neutrophil chemotaxis was disrupted, causing inappropriate extravasation of neutrophils into various organs. Tissue injury inflicted by this process leads to multiorgan failure (15); which is the salient clinical feature that differentiates sepsis from infection by Sepsis-3 definition (1).

In contrast to our findings on CXCR2 surface level, our study fails to demonstrate the changes of CD64 and CCR2 surface levels as sepsis-related. CD64 and CCR2 receptors are not constitutively expressed on neutrophils at resting state (15, 25); however, earlier studies showed that CD64 and CCR2 surface levels were increased during sepsis (3). The amount of CD64 mRNA elevates to a measurable level within 1–3 h of the inciting events leading to a significant increment of CD64 surface level within 4–6 h (13). The emergence of CD64 surface receptors was thought to enhance neutrophil phagocytic activity through its high affinity to Fcγ part of IgG (26). Our study found that, even though a significant difference in CD64 surface level between septic shock and infection groups were observed during Day 2 to Day 5, this is mainly because CD64 surface level in infected patients plummets after Day 1 as opposed to septic shock patients whose CD64 surface level gradually decrease. At the onset of the disease, however, CD64 surface level from septic shock and infected patients are within the same range. Also, as shown by the regression coefficients, both infection and septic shock status could predict the degree of CD64 surface level over time despite the stronger effect exerted by septic shock. These findings suggest that a between-group difference in CD64 surface level likely stems from a more rapid recovery in non-sepsis patients who have milder infection compared to septic shock patients who have a more severe infection. In other words, CD64 surface level is not explicitly altered by sepsis but rather have a dose-response relationship with infection. This hypothesis is also supported by many studies which showed the rise of CD64 surface level as a marker of bacterial infection on a background of various other conditions, such as critically-ill status (27, 28), postoperative period (10, 29), and autoimmune diseases (30). Furthermore, previous studies showed that peripheral blood neutrophils from mice, which developed sepsis from cecal ligation and puncture, express high amount of CCR2 mRNA and CCR2 surface level as well as marked chemotaxis in response to CCL2 (14, 31). Since these mice were also had multiple organ dysfunctions and neutrophil accumulations in heart, lung, and kidney, the presence of CCR2 surface receptors was implicated in driving neutrophil infiltration to distant organs during sepsis (14). Nonetheless, our study found that CCR2 surface levels were similar between septic shock and infection groups and, like CD64 receptors, both infection and septic shock status could predict CCR2 surface levels over time. These findings suggest that elevation of CCR2 surface level is likely to be related to infection rather than sepsis. Organ dysfunction found in the earlier study may be attributable to decreased CXCR2 surface level, which was also demonstrated in these mice (14). Besides, CCR2 surface expression on neutrophil is not specific to sepsis or infection and can be found in other conditions, such as rheumatoid arthritis (32), and ischemic liver injury (33).

With regard to apoptosis and NETosis, although current evidence associates apoptosis delay and increased NETosis activity with sepsis, our study cannot confirm that they are sepsis-related. We found that, even if apoptotic activity starts to rise toward the end of the week, apoptotic activity of neutrophils from septic shock and infected patients was similar throughout the study period. Neither infection nor septic shock status could predict the percentages of apoptosis over time. Hence, within the timeframe of this study, apoptosis is less likely to contribute to the sequelae of sepsis. Similar to apoptosis, NETosis activity increases later during the clinical course. If we compare NETosis activity in septic shock and infected patients on a day-by-day basis, we might have to conclude that NETosis is suppressed in septic shock patients on Day 3. In fact, a few earlier longitudinal studies in sepsis which examined peripherally-measured NETosis for seven days also suggested that NETosis was suppressed throughout the clinical course of sepsis compared to healthy controls (22, 34). Nonetheless, we believe the different between septic shock and infected patients may concern the day NETosis spikes rather than the level of NETosis activity between infected and septic shock patients on the same day. In infection group, NETosis activity peaks on Day 3 and gradually decline thereafter. In septic shock groups, NETosis activity is highest on Day 14. Possibly, we may find a peak-and-fall pattern of NETosis activity in septic shock patients as we observed in infected patients if we follow the patients for a longer period. Despite the absence of statistical significance, patients who recovered at Day 14 seems to have the highest NETosis activity compared to patients who are still critically-ill or deceased at Day 14. Thus, the rise of NETosis in peripheral blood may signify the recovery of from infection; infected patients who generally recover quicker than sepsis patients, therefore, experience the NETosis spike before sepsis patients.

In summary, by using infection as control, we demonstrated that the relationship between each neutrophil function and sepsis is unique and possibly reflects sepsis processes from different angles. CXCR2 surface level is related to sepsis activity while CD64 and CCR2 surface levels link to sepsis *via* infection. Peripherally-measured NETosis may signify the recovery from infection as it elevates during the less dynamic phase of the disease. Apoptosis during the study period may be equally affected by both sepsis and infection since its activity is indistinguishable between sepsis and infection. The strengths of our study are as follows. Firstly, data from our study can be applied to the current practice since we define septic shock and infection as per the latest clinical definition. Secondly, in contrast to previous studies which focused mainly on the magnitude of neutrophil functions, we provide data in a longitudinal fashion in relation to the clinical progression of the patients. Lastly, we examined the neutrophil functions not only by the group-averaged values but also with multilevel regression model which take into account the interindividual variation of the response pattern during analysis. We believe both methods should be used simultaneously since they serve different but equally important purposes. Group-averaged values give more insight on the neutrophil functions on the specific days in patients stratified by one characteristic (e.g., sepsis status, clinical status) and point out the one with the association which is strong enough to stand out even in the presence of unadjusted interindividual variation. However, multilevel model better highlight associations between neutrophil functions and variable of interest, especially in the neutrophil functions expressing a high degree of interindividual variation in the response pattern such as CCR2 surface level. Limitations of our study concern an inability to apply the result to patients with pre-existing immunological aberrations (e.g., cancer, autoimmune diseases, post-surgery). Further studies in a more immunologically heterogenous patients, especially for CXCR2 surface level, should be done to examine the generalizability of our observation. In addition, we measured these neutrophil functions from circulating pools of neutrophils; whether tissue neutrophils have the same or different phenotype is outside of the scope of our study.

## Conclusion

With infection as control, a reduction of CXCR2 surface level is associated with sepsis; its level can be used to distinguish sepsis from infection at the onset of the disease. CD64 surface level, CCR2 surface level, and NETosis are not directly sepsis-related; their relationship with sepsis is instead mediated by the effect of infection. Apoptotic activity in septic shock patients does not found to be delayed compared to patients with infection.

## Data Availability Statement

The raw data supporting the conclusions of this article will be made available by the authors, without undue reservation.

## Ethics Statement

The studies involving human participants were reviewed and approved by Human Research Ethics Committee of the Faculty of Medicine, Prince of Songkla University, Thailand. The patients/participants provided their written informed consent to participate in this study.

## Author Contributions

All authors took part in the conceptualization and methodology planning of the study. CS and RN did the validation and conduction of study experiments. CS, PV, and RN were responsible for data curation and formal analysis of the study. The original draft was prepared by CS and reviewed by RN, PV, and BK. All authors have read and agreed to the published version of the manuscript. BK oversaw the project administration as well as funding acquisition. All authors contributed to the article and approved the submitted version.

## Funding

This study was financially supported by a research grant from the Faculty of Medicine, Prince of Songkla University (Grant No. REC 61-090-14-1).

## Conflict of Interest

The authors declare that the research was conducted in the absence of any commercial or financial relationships that could be construed as a potential conflict of interest.
